# Mechanism of quercetin therapeutic targets for Alzheimer disease and type 2 diabetes mellitus

**DOI:** 10.1038/s41598-021-02248-5

**Published:** 2021-11-25

**Authors:** Guoxiu Zu, Keyun Sun, Ling Li, Xiuli Zu, Tao Han, Hailiang Huang

**Affiliations:** 1 Department of Traditional Chinese Medicine, Shandong University of Traditional Chinese Medicine, Jinan, 250355 China; 2 Department of Pharmacy, Shandong University of Chinese Medicine, Jinan, 250355 China; 3 Graduate Office, Shandong University of Traditional Chinese Medicine, Jinan, 250355 China; 4 Department of Rehabilitation Medicine, Shandong University of Traditional Chinese Medicine, Jinan, 250355 China

**Keywords:** Plant sciences, Endocrinology, Medical research

## Abstract

Quercetin has demonstrated antioxidant, anti-inflammatory, hypoglycemic, and hypolipidemic activities, suggesting therapeutic potential against type 2 diabetes mellitus (T2DM) and Alzheimer’s disease (AD). In this study, potential molecular targets of quercetin were first identified using the Swiss Target Prediction platform and pathogenic targets of T2DM and AD were identified using online Mendelian inheritance in man (OMIM), DisGeNET, TTD, DrugBank, and GeneCards databases. The 95 targets shared among quercetin, T2DM, and AD were used to establish a protein–protein interaction (PPI) network, top 25 core genes, and protein functional modules using MCODE. Metascape was then used for gene ontology and kyoto encyclopedia of genes and genomes (KEGG) pathway enrichment analysis. A protein functional module with best score was obtained from the PPI network using CytoHubba, and 6 high-probability quercetin targets (AKT1, JUN, MAPK, TNF, VEGFA, and EGFR) were confirmed by docking simulations. Molecular dynamics simulation was carried out according to the molecular docking results. KEGG pathway enrichment analysis suggested that the major shared mechanisms for T2DM and AD include “AGE-RAGE signaling pathway in diabetic complications,” “pathways in cancer,” and “MAPK signaling pathway” (the key pathway). We speculate that quercetin may have therapeutic applications in T2DM and AD by targeting MAPK signaling, providing a theoretical foundation for future clinical research.

## Introduction

According to the 2017 global burden of disease study, type 2 diabetes mellitus (T2DM) and Alzheimer’s disease (AD) are the sixth and tenth leading causes of premature death worldwide^1^, and many studies have found strong epidemiological, pathological, and clinical associations between T2DM and AD^2^. The incidence of AD is about twofold greater in T2DM patients than in non-diabetic individuals, while a disproportionate number of AD patients have a history of early or clinical diabetes. Diabetes and AD also share numerous common risk factors, including metabolic disease (obesity), cardiovascular disease, older age, and progressive insulin resistance^2^, as well as common pathogenic processes such as chronic hyperglycemia, tissue amyloid β (Aβ) deposition and toxicity, cellular oxidative stress, and chronic inflammation.

Indeed, the term “type 3 diabetes” has been proposed to describe AD based on the hypothesis that AD-associated dementia is caused in part by insulin resistance and insufficient insulin-like growth factor signaling in the brain^3^. It is believed that endocrine and metabolic diseases characterized by elevated blood glucose due to insulin resistance and death of insulin-producing pancreatic islet β-cells can lead to chronic cardiovascular diseases and ultimately to AD. Alzheimer’s disease is characterized by the accumulation of Aβ in the brain parenchyma (termed senile plaques, SPs) and abnormal hyperphosphorylation of the intermediate filament protein tau within neurons, forming neurofibrillary tangles (NFTs). The presence of these inclusions results in synaptic loss, particularly in neocortex and hippocampus, oxidative stress, and neuroinflammation, ultimately leading to neuronal dysfunction and death^4^. Tau protein and human islet amyloid polypeptide (hIAPP) deposits are also found in the pancreatic islets of T2DM patients. Moreover, pharmacological studies have shown that some traditional hypoglycemic drugs can slow the progression of AD^4^.

While there is strong evidence for an association between T2DM and AD at multiple levels, the seminal molecular mechanisms linking these disorders remain unknown. One potential strategy for identifying these molecular linkage pathways is to screen for common drug targets, which would also provide new possibilities for the treatment of T2DM and AD.

Quercetin (3, 3′, 4′, 5, 7-pentahydroxyflavone) is a natural polyhydroxyflavonoid found in the flowers, leaves, and fruits of edible plants such as onion, apple, lettuce, and cabbage^4^. In addition, many Chinese herbal medicines such as Sophora japonica, Ginkgo biloba leaves, Hypericum perforatum, and cortex moutan are enriched in quercetin^5^. Numerous studies have documented bioactivities of quercetin relevant to T2DM and AD pathology, such as antioxidant, anti-inflammatory, hypoglycemic, and hypolipidemic effects. Quercetin has also been shown to reduce blood glucose concentration and preserve islet function, insulin sensitivity, and β-cell numbers in diabetic model rats and mice^6,7^.

At present, there is no broadly effective drug treatment to delay AD onset, slow progression, or improve outcome^8^, and the few approved mediations can only transiently improve symptoms^9^. Also, many synthetic drugs with antioxidant, anti-inflammatory, hypoglycemic, and (or) hypolipidemic activities induce adverse events that preclude clinical use. The use of natural substances to treat neurodegenerative diseases such as AD and metabolic diseases such as T2DM is a promising alternative as such agents are often easily and inexpensively isolated and have well documented biomechanisms and safety profiles. Yao et al. conducted a cross-sectional study of nearly 20,000 people^10^. The results show that there is a significant negative correlation between dietary quercetin intake and the prevalence of T2DM in Chinese population, which suggests that dietary quercetin intake may play a positive role in the prevention and treatment of T2DM, such as anti-oxidation, anti-inflammatory, blood sugar and blood lipid. At the same time, it is supported by experiments^7^, Supplementation of quercetin can reduce the blood glucose concentration and promote islet in diabetic rats. β Cell recovery and increased insulin release. Quercetin has insulin sensitizing effect. Long term low-dose dietary supplementation can reduce insulin resistance (IR)^6^ in diabetic mice. Quercetin and its glycosides have extensive neuroprotective effects against AD, and its mechanism mainly includes interference β-the formation and deposition of starch protein can inhibit the hyperphosphorylation of tau protein to intervene the disease development process of AD. It can play a protective role on nerve cells through anti-inflammatory response, antioxidant stress and inhibition of apoptosis. In addition, it also has estrogen like effect on nerve cells. In recent years, the estrogen like neuroprotective effect of quercetin has attracted more and more attention. Epidemiology found that the probability of ad in postmenopausal women is much higher than that in men, suggesting that estrogen reduction is a risk factor for AD, and estrogen replacement therapy may prevent and delay the occurrence of AD. Quercetin has also shown anti-dementia and neuroprotective efficacies in models of dementia/AD and ischemia–reperfusion injury by mitigating neuronal oxidative stress and neuroinflammation, and by improving calcium homeostasis, growth factor signaling, and neuroplasticity, ultimately preventing neuronal apoptosis^11–15^. Further, a number of large double-blind clinical studies have demonstrated that quercetin can reduce the composite end-point of death in patients with T2DM and AD. In the present study, we used a systematic bioinformatics approach (network pharmacology) and molecular docking simulations to identify quercetin-binding targets that may also be potential therapeutic targets for AD and T2DM.

## Materials and methods

### Screening of potential quercetin targets

The Traditional Chinese Medicine Systems Pharmacology and Analysis Platform (TCMSP) was used to search for preparations with “quercetin” as a key word, and results were then combined with the Chinese Medicine Encyclopedia database (ETCM) to find Chinese herbal medicines enriched in quercetin. Quercetin targets were then identified using the PubChem database with “quercetin” as the key word. Based on the notion that drugs of similar structure will have common (overlapping) targets, five potential targets of quercetin were predicted using Swiss Target Prediction (http://www.swisstargetprediction.ch/)^16^ with species “Homo sapiens” and a probability > 0.1 as the selection criteria. Target names were standardized according to Uniprot protein database terminology (https://www.uniprot.org/)^17^.

### Screening of quercetin targets for T2DM and AD treatment

Pathogenic molecules contributing to T2DM and AD was screened using Online Mendelian Inheritance in Man (OMIM, https://omim.org/)^18^ TTD (http://db.idrblab.net/ttd/), DisGeNET (https://www.disgenet.org/), DrugBank (https://www.drugbank.ca/)^19^, and GeneCards (https://www.genecards.org/)^20^ using the terms “Type 2 diabetes mellitus” and “Alzheimer’s Disease.” By intersecting quercetin targets with disease-associated molecules targets using a Venn diagram, we obtained targets potentially relevant to T2DM and AD treatment. Cytoscape software^21^ was then used to map the component–target–disease network as described in detail below.

### Construction of protein–protein interaction (PPI) network

To identify interaction targets of quercetin potentially relevant to T2DM and AD pathology and treatment, we used the online drawing tool Interactive Venn (http://www.interactivenn.net/)^22^ to construct a Venn diagram. The overlapping targets were then uploaded to the STRING11.0 platform (https://string-db.org/)^23^ to obtain a protein–protein interaction (PPI) network. The maximal clique centrality (MMC) algorithm in the CytoHubba plug-in was used for comprehensive analysis of network topology to obtain the top 25 hub genes^23^. Then MCODE^24^, a cluster analysis plug-in for Cytoscape3.6.0 (https://cytoscape.org/), was utilized to further analyze the PPI network. The targets in the protein functional module with best MCODE score were selected as core targets for further molecular docking simulations.

### Enrichment analysis

Gene Ontology (GO) functional analysis and Kyoto Encyclopedia of Genes and Genomes (KEGG) pathway enrichment analysis were performed using Metascape (https://metascape.org/)^25^, with *p* < 0.01 as the cutoff criterion. The top 10 GO items and top 20 KEGG pathways that meet this criterion were visualized using OmicShare (http://www.omicshare.com/) and microscopic letter (http://www.bioinformatics.com.cn).

### Construction of a component–target–pathway network

Cytoscape3.6.0 was used to construct a component–target–pathway network based on KEGG enrichment analysis, and the topological parameters of the network were analyzed using the built-in Network Analyzer tool.

### Molecular docking verification

The PDB formats of the core target proteins were downloaded from the RCSB PDB database (http://www.rcsb.org/). We used PyMol 2.4 software to remove water molecules and separate the original ligand from the core target protein, and then imported the processed protein targets into AutoDock Tools 1.5.6 software^26^ for hydrogenation, calculation of total charge, and setting the atomic type. Results were saved in “pdbqt” format. The mol2 structures of the core targets were downloaded from the TCMSP database, and AutoDock Tools was used to set the rotatable bonds. Files were again saved in “pdbqt” format and then imported to AutoDock Vina to perform molecular docking. Finally, PyMol software was used to visualize the docking results and establish the docking interaction pattern.

### Molecular dynamics simulation

**Figure 1:**
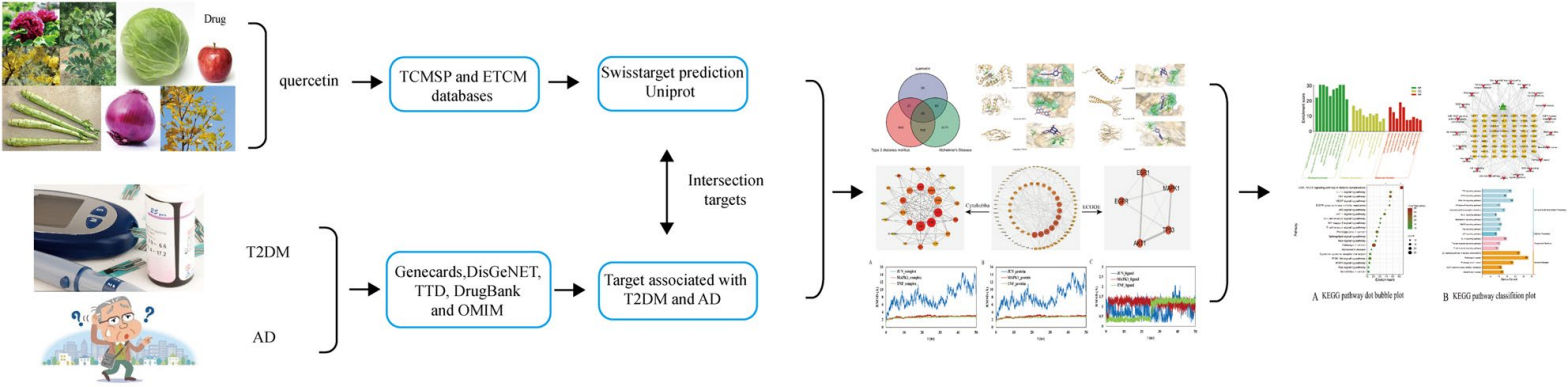
Flowchart of the network pharmacology study investigating quercetin targets for T2DM and AD treatment.

Amber14 was used for molecular dynamics simulation. Amber-99sb force field was used for protein system, gaff force field was used for quercetin molecule, TIP3P water molecule model and periodic boundary were used. All systems were first optimized, temperature rise and equilibrium, and then 50 ns molecular dynamics simulation was carried out by sander. Take the trajectory of 10–50 ns and calculate the binding energy between quercetin and protein using mm/pbsa.py. Analyze the trajectory using the cpptraj module in ambertools. A flowchart of the network pharmacology study is shown in Fig. 1.

## Results

### Candidate quercetin targets for T2DM and AD treatment

**Figure 2:**
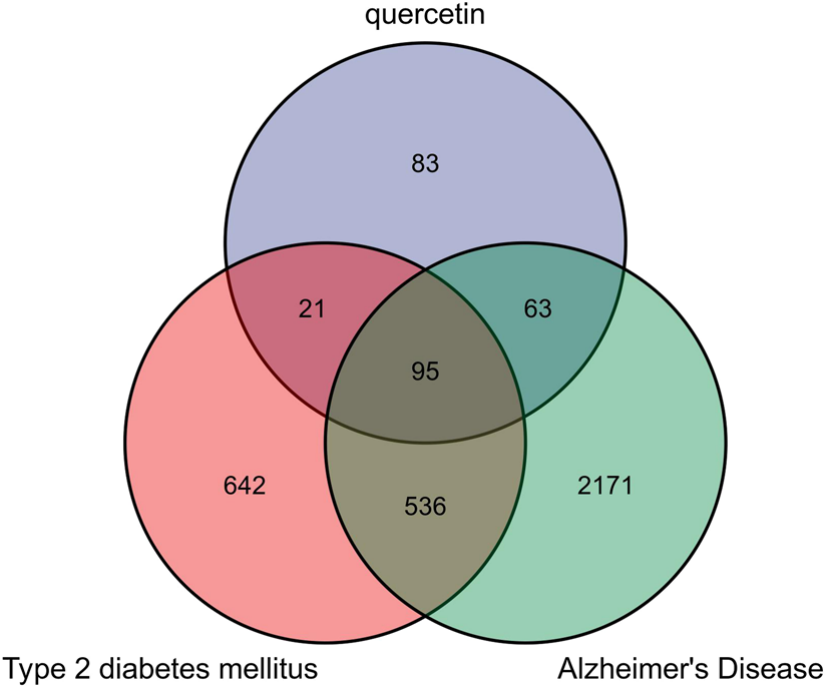
Venn diagram showing the overlap among quercetin targets (blue), T2DM-related gene products (red), and AD-related gene productions (green). A total of 95 gene products were identified as common.

After screening the literature and online databases, we identified 263 candidate gene products potentially targeted by quercetin. Screening for T2DM pathogenic targets retrieved 30 genes from OMIM, 175 from TTD, one from DisGeNET (with relevance score > 0.1), 266 from DrugBank, and 1108 from GeneCards (relevance score > 1). For AD, we retrieved 9 gene products from OMIM, 143 from TTD, 218 from DisGeNET, 67 from DrugBank, and 2712 from GeneCards. After removing duplicates, 1294 T2DM-related and 2865 AD-related genes were included in subsequent analyses. To obtain quercetin targets with relevance to T2DM and AD pathology and treatment, we constructed a Venn diagram, and a total of 95 overlapping targets were obtained (Fig. 2).

### PPI network of quercetin targets for T2DM and AD treatment

**Figure 3:**
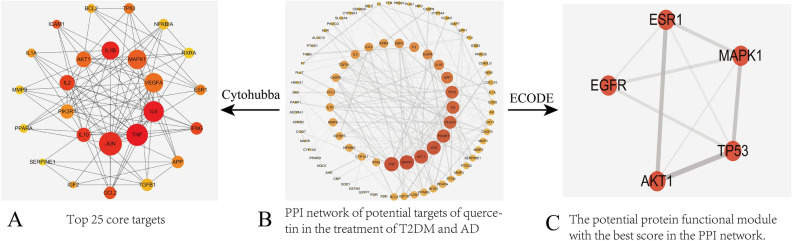
PPI network.

To estimate the contributions of these candidate common targets to disease and reveal the interactive effects, all 95 were imported into the STRING database with highest confidence set at 0.9 to obtain a PPI network. The PPI network results were saved in TSV text format and imported into Cytoscape3.6.0 for network topology analysis to identify the top 25 targets (arranged in descending order of relevance as a concentric circle in Fig. 3A). The PPI network (Fig. 3B) includes 273 edges among the 95 nodes (circles), where edge thickness represents the interaction strength. To further refine the list of quercetin targets relevant to T2DM and AD treatment, we conduct cluster analysis on the PPI network based on shared mechanisms using MCODE. This analysis yielded a function module of five key nodal proteins (Fig. 3C). It is generally believed that proteins in such functional modules are more closely related and may interact to perform specific biological functions. Therefore, these proteins may have important regulatory effects on T2DM and AD.

### Possible molecular targets of quercetin and associated regulatory signaling pathways relevant to T2DM and AD treatment

**Figure 4:**
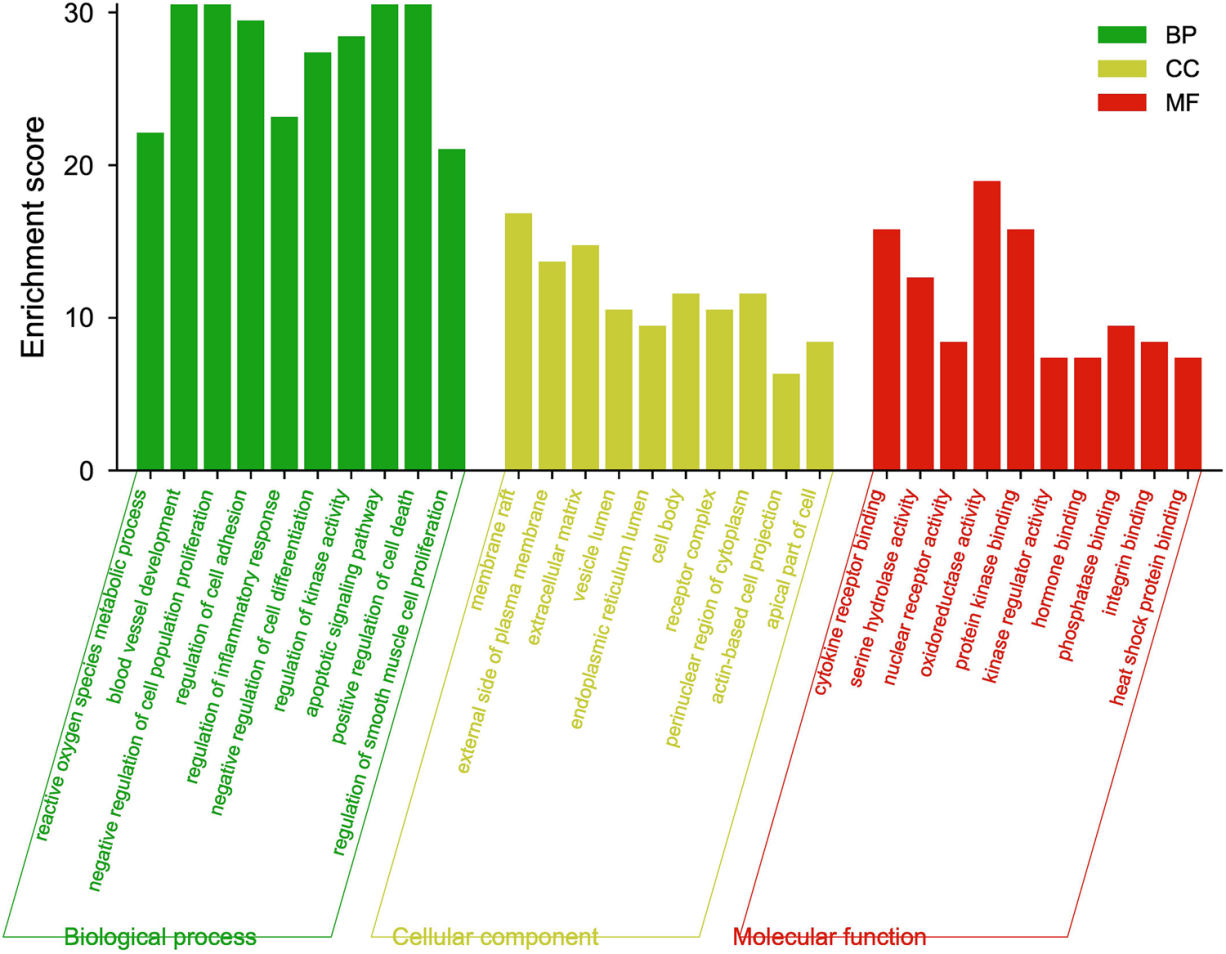
GO functional analysis of quercetin targets relevant to T2DM and AD treatment.

**Figure 5:**
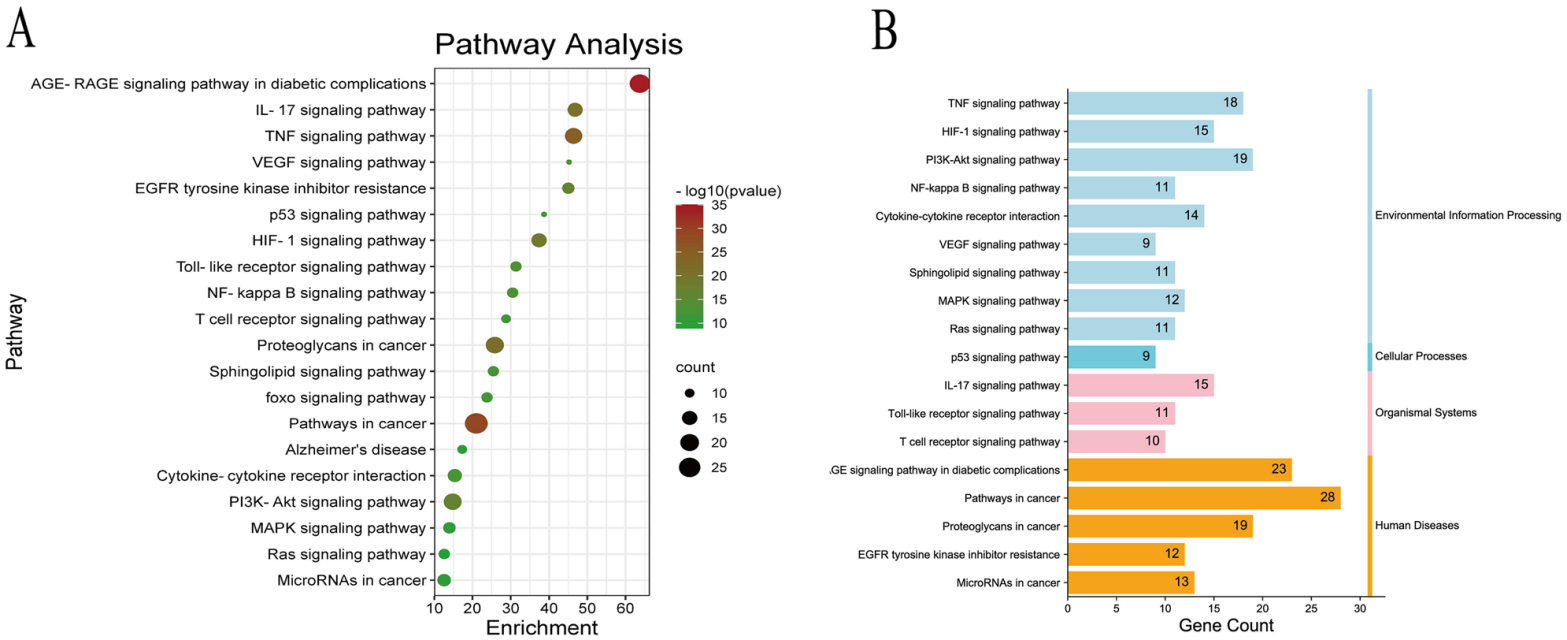
KEGG pathway enrichment analysis of quercetin targets relevant to T2DM and AD treatment. (**A**) KEGG pathway dot bubble plot. (**B**) KEGG pathway classification plot.

We further conducted GO functional analysis and KEGG pathway enrichment analysis^27–29^ to elucidate the biological effects of quercetin targets and related signaling pathways relevant to T2DM and AD treatment. The top 10 GO items was selected based on *p* values (Fig. 4). Similarly, the top 20 KEGG pathway items were selected based on *p* values (Fig. 5A) and arranged according to category (Fig. 5B).

The common targets were enriched in molecules with GO molecular function terms “cytokine receptor binding,” “serine hydrolase activity,” “oxidoreductase activity,” “protein kinase binding,” and “antioxidant activity,” biological processes terms “positive regulation of cell apoptosis” (GO:0097190, GO:0010942, GO:0008285), “regulation of inflammatory response” (GO:0050727), “regulation of reactive oxygen species metabolic process” (GO:0072593), “cellular response to external stimulus” (GO:0071496), and “regulation of protein serine/threonine kinase activity” (GO:0071900), and cellular components terms “membrane raft,” “external side of plasma membrane,” “extracellular matrix,” “vesicle lumen,” “endoplasmic reticulum lumen,” “cell body,” “receptor,” “perinuclear region of cytoplasm,” “actin-based cell projection,” and “apical part of cell.” According to KEGG pathway enrichment analysis, most of these common targets are involved in “MAPK signaling pathway,” “TNF signaling pathway,” “AGE-RAGE signaling pathway in diabetic complications,” “pathways in cancer,” “proteoglycans in cancer,” “interleukin (IL)-17 signaling pathway,” “hypoxia-inducible factor (HIF)-1 signaling pathway,” “PI3K-Akt signaling pathway,” “EGFR tyrosine kinase inhibitor resistance,” “Toll-like receptor signaling pathway,” “NF-kappa B signaling pathway,” “cytokine-cytokine receptor interaction,” “VEGF signaling pathway,” and (or) “Alzheimer’s disease.” Thus, the common targets of quercetin, T2DM, and AD are strongly enriched in “MAPK signaling pathway” components as indicated by four of the MCODE nodes (Fig. 3C) and two of the top 10 GO molecular function items. In addition, most of the biological processes items are associated with MAPK activity, such as “kinase activity regulation,” “cell proliferation,” and “apoptosis,” suggesting that the MAPK signaling pathway is the core quercetin target relevant to T2DM and AD treatment.

### Component-target-pathway network construction

**Figure 6:**
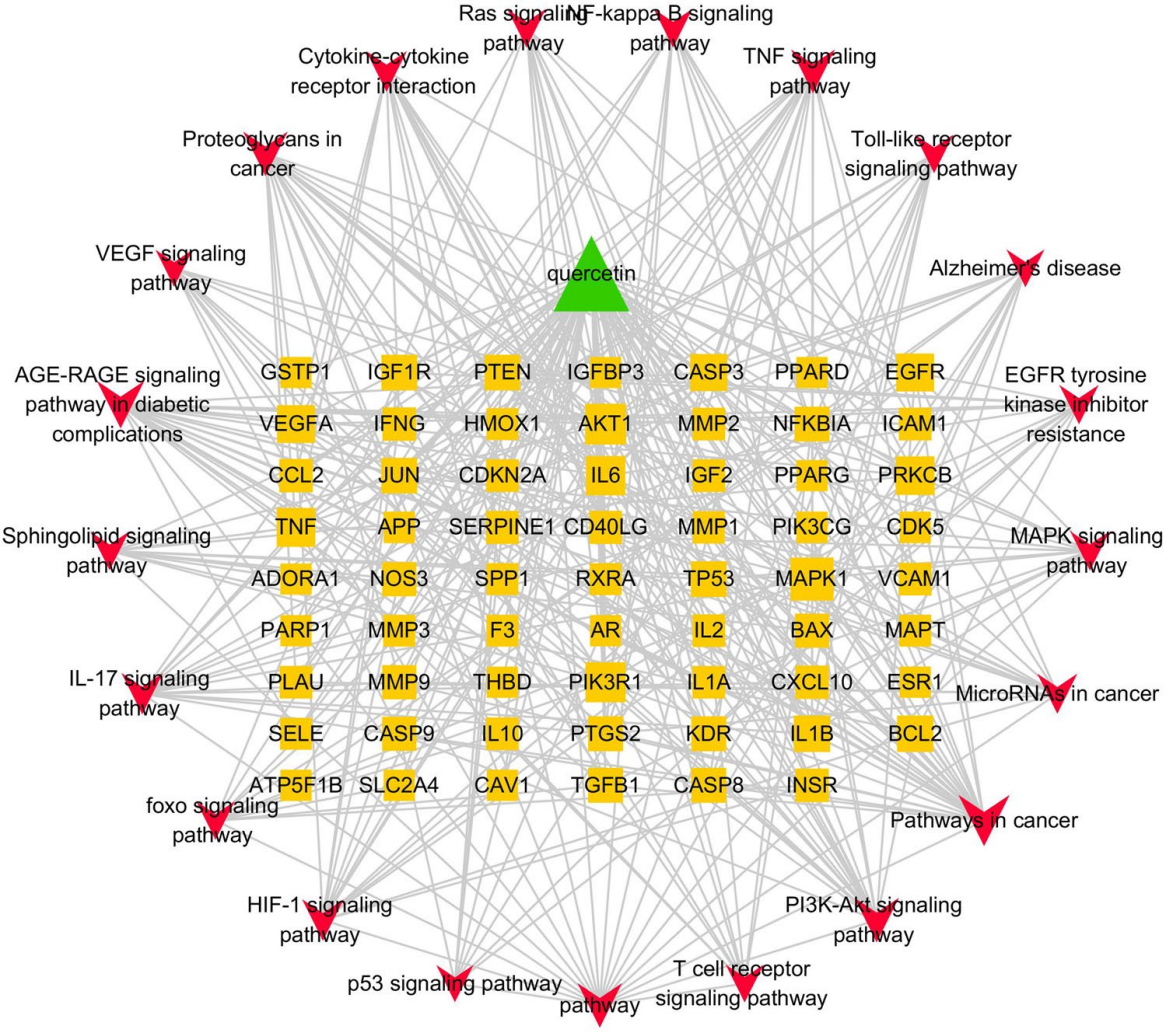
Component–target–pathway network for quercetin targets relevant to T2DM and AD treatment.

A component-target-pathway network was constructed based on KEGG pathway enrichment analysis using Cytoscape3.6.0 (Fig. 6) in which the importance of a given node is indicated by the number of connections to other nodes. The average degree of importance (average number of connection to a given node) was 8.64, and there were 11 targets with degrees higher than 8.64: MAPK1 (degree = 18), AKT1 (degree = 15), PIK3R1 (degree = 14), IL6 (degree = 13), PRKCB (degree = 12), TNF (degree = 12), VEGFA (degree = 11), EGFR (degree = 11), CASP3 (degree = 10), BCL2 (degree = 9), and IL1B (degree = 9). Since MAPK1, AKT1, and EGFR were also included in the best scoring protein functional module, we speculate that these may be the key targets of quercetin for T2DM and AD treatment.

### Molecular docking results analysis

**Figure 7:**
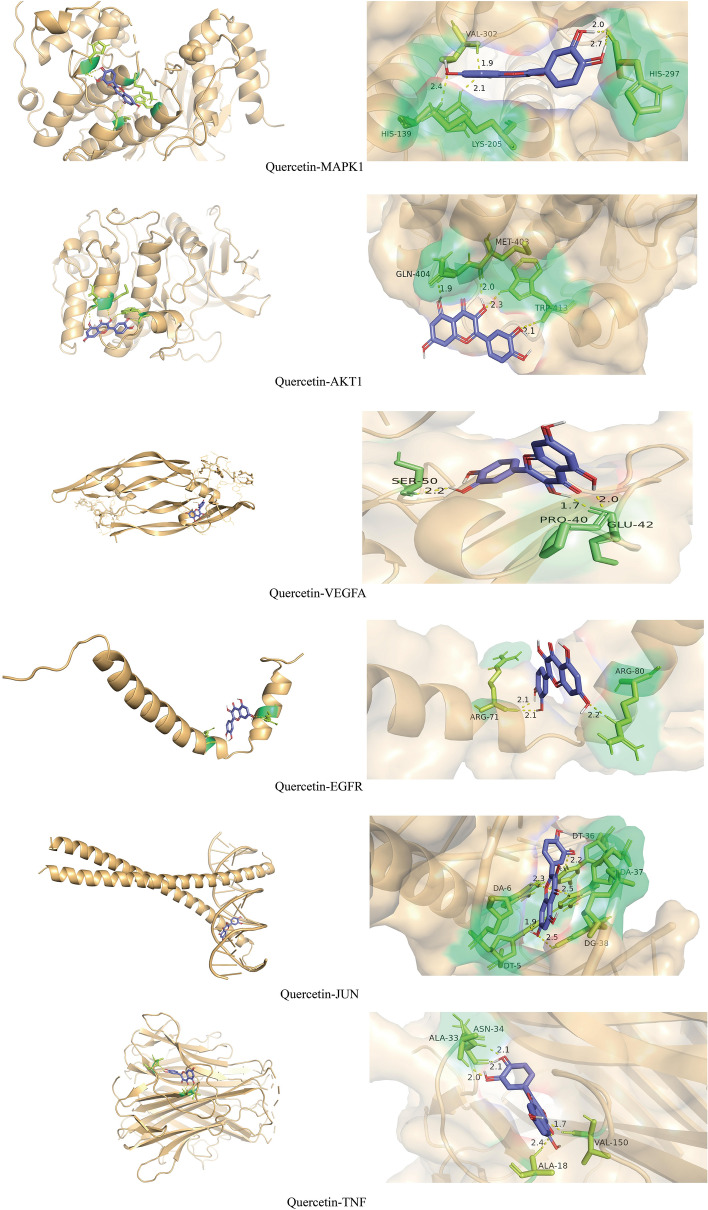
Molecular docking diagrams of quercetin binding to (from top to bottom) MAPK1, AKT1, VEGFA, EGFR, JUN, and TNF.

Finally, the interactions with core proteins in the PPI, MCODE, and component-target-pathway networks were verified by molecular docking simulations. The most likely and stable molecular interactions are generally those with lower energy after binding. Six core molecules were tested in docking simulations (Table 1 and Fig. 7), and all showed good binding affinity with an average free energy change of − 6.47 kcal/mol. For the quercetin–MAPK1 docking model, the small molecule ligand dapagliflozin potentially fit into the interface pocket formed by interacting amino acid residues in the protein. As shown in Fig. 7, a hydrogen bond was formed between quercetin and VAL302, HIS297, HIS139, and LYS205 near the active site of MAPK1.

**Table 1:** Binding energy of quercetin with MAPK1, AKT1, VEGFA, EGFR, JUN, and TNF.

Bioactive component	Target name	PDB ID	Binding energy (kcal/mol)
Quercetin	MAPK1	5K4I	− 6.28
	AKT1	2UZR	− 5.94
	VEGFA	6D3O	− 5.81
	EGFR	3POZ	− 5.76
	JUN	5T01	− 6.64
	TNF	7ATB	− 8.4

PDB ID: Protein Data Bank Identification Code.

### Molecular dynamics simulation results

After molecular docking, three proteins with good binding were selected according to the docking results, and the sander module in amber was used for 50 ns molecular dynamics simulation to analyze the stability of the system. The binding energy data of quercetin molecule and target protein were obtained by mm/GBSA calculation. After 50 ns molecular dynamics simulation, the structures of MAPK1 and TNF proteins are relatively stable, and JUN protein has certain structural changes. It is speculated that it is mainly due to the combination of JUN and DNA double strand in the original crystal structure to form a complex. This simulation only studies the combination of JUN and quercetin molecules, and deletes the DNA double strand, resulting in certain structural changes in the region where JUN binds to DNA.

**Figure 8:**
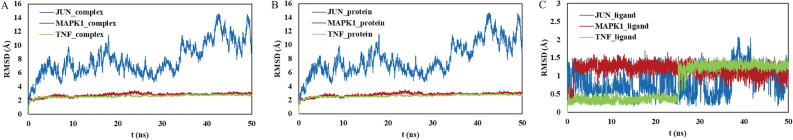
RMSD plot during molecular dynamics simulations. (**A**) The RMSD of protein–ligand complex during MD simulation. (**B**) The RMSD of protein during MD simulation. (**C**) The RMSD of quercetin during MD simulation.

On the whole, the binding of quercetin to the three proteins was relatively stable, and remained at the predicted binding site of molecular docking in the process of molecular dynamics simulation. Only certain conformational changes were observed in TNF system. The binding energies of quercetin molecule and three target proteins were further calculated by mm/GBSA method. The results showed that the binding energy between quercetin and TNF was the strongest, in fact MAPK1, followed by JUN. below is the binding energy (Table 2) and molecular dynamics simulation diagram (Fig. 8).

**Table 2:** The calculated binding energy of quercetin against JUN, MAPK1 and TNF.

Protein	Binding energy (kcal/mol)
JUN	− 12.5549 ± 3.4356
MAPK1	− 25.4414 ± 4.0935
TNF	− 40.1137 ± 3.7513

## Discussion

Type 2 diabetes is the third most common non-infectious disease in the world. It is characterized by poor blood glucose control due to insulin resistance and progression apoptotic death of insulin-producing islet β-cells^30^. As T2DM progresses, the loss of insulin production creates an absolute insulin deficiency that can only be addressed by exogenous replacement. Therefore, enhancing insulin sensitivity and reducing β-cell apoptosis are the ultimate strategies for the prevention and treatment of T2DM^31^. Several studies have found that quercetin can reduce blood glucose, peripheral insulin resistance, and indicators of oxidative stress induced by high glucose in rats, a response profile similar to the widely used antidiabetic drug metformin.

Alzheimer’s disease is the most common neurodegenerative disorder. Clinically, it is characterized by progressive cognitive impairment, memory loss, and marked changes in personality and behavior. The two primary hypotheses proposed to explain the progressive neurodegeneration and neurological impairments of AD are cytotoxic β-amyloid deposition (plaque formation) due to abnormal processing and hyperphosphorylation of tau as these are the pathological hallmarks of the AD brain. In turn, these plaques and NFTs may cause oxidative stress and inflammation, leading to synaptic failure, neuronal apoptosis (especially basal forebrain cholinergic neurons and various hippocampal and cortical neurons), and brain atrophy^32,33^. These pathomechanisms are not mutually exclusive and indeed may interact, creating pathogenic cascades. Thus, many protein signaling pathways and gene regulation pathways may contribute to the pathological changes in AD.

Many natural components found in traditional herbal preparations have documented antioxidant, anti-inflammatory, antidiabetic, and anti-apoptotic activities, suggesting potential utility for AD and T2DM treatment. For instance, quercetin can improve insulin production by rat insulinoma INS-1 cells in response to high glucose, as well as downregulate the relative expression levels of pro-apoptotic Bax and PDX-1 mRNAs, inhibit oxidative stress caused by H_2_O_2_, and reduce INS-1 cell apoptosis^34^. Quercetin can also clear reactive oxygen species (ROS) and reactive nitrogen species and inhibit the level of low density lipoprotein peroxidase, thereby reducing the incidence of cardiovascular disease^35^. Further, quercetin was shown to reduce ischemic injury and peroxynitrate injury by inhibiting nitric oxide synthase and xanthine dehydrogenase activities^36^.

The current study revealed several potential molecular mechanisms for AD and T2DM as well as possible treatment targets by combining network pharmacology with molecular docking. By integrating and collating information from several databases, we identified 95 potential quercetin targets involved in T2DM and AD pathology, and further defined a potential protein functional module of five proteins and 25 core genes from the PPI network. The biological processes mediated by these functional module proteins include protein kinase signaling and cell proliferation, consistent with pathogenic pathways of T2DM and AD identified in previous studies.

CytoHubba and MCODE identified AKT1, JUN, MAPK, TNF, VEGFA, and EGFR as core targets of quercetin that may contribute to AD and T2DM. The PI3K/Akt signaling pathway is anti-apoptotic, and AKT1 inhibits Akt phosphorylation (activation), which in turn upregulates the activity of GSK3β, a kinase implicated in the pathogenesis of both T2DM and AD. JUN is a member of the mitogen-activated protein kinase (MAPK) family implicated in the onset, progression, and reversal of vascular diseases through modulation of vascular cell protein expression, proliferation, oxidative stress, and apoptosis. Downregulation of JUN can significantly reduce the expression of inflammatory factors and inhibit endothelial cell apoptosis^37^. Tumor necrosis factor (TNF) is a multifunctional cytokine that can directly damage islet β-cells and induce insulin resistance by inhibiting the transduction of insulin signals. MAPK is a serine/threonine protein kinase involved in cell proliferation, differentiation, apoptosis, and inflammatory responses^38^. Activation of MAPK can also increase tau phosphorylation in neurons^39^, while inhibition of MAPK expression in hippocampus can significantly improve memory, cognitive function, synaptic plasticity, and neuronal metabolism^40^. Suppression of MAPK can also improve insulin resistance by upregulating the expression of the insulin-dependent glucose transporter GLUT-4 in striatal muscle and adipose tissue, and by enhancing the sensitivity of insulin receptors, thereby promoting the absorption and utilization of glucose. Therefore, MAPK may be a core target for the treatment of T2DM and AD^41^.

**Figure 9:**
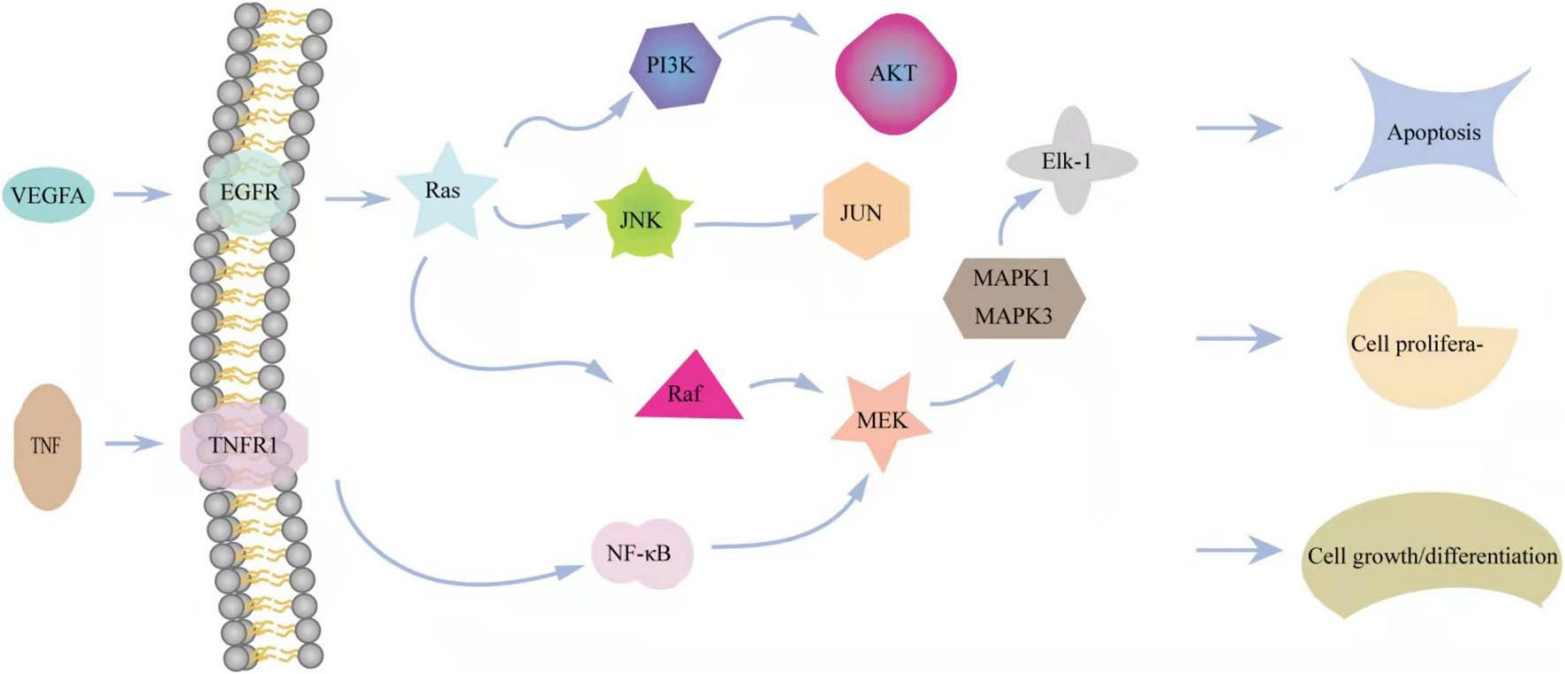
Components of MAPK signaling pathway influenced by quercetin.

To better understand the interactions among these target genes, we conducted GO and KEGG pathway analyses. The target genes were mainly related to cell apoptosis, regulation of inflammatory response, regulation of ROS, metallic process, and other biological processes. KEGG pathway enrichment analysis revealed that target proteins are involved in the MAPK, TNF, HIF-1, IL-17, PI3K, NF-kappa B, Toll-like receptor, and VEGF signaling pathways, AGE-RAGE signaling pathway in diabetic complications, pathways in cancer, proteoglycans in cancer, EGFR tyrosine kinase inhibitor resistance, cytokine–cytokine receptor interaction, and Alzheimer’s disease. We hypothesize that quercetin may play a therapeutic role in T2DM and AD through the regulation of these pathways, but especially through modulation of MAPK signaling (Fig. 9).

A variety of extracellular signaling molecules (growth factors, neurotransmitters, cytokines, hormones, etc.) stimulate MAPK signaling via the MAPKKK–MAPKK cascade, ultimately regulating both cytoplasmic processes and gene expression pathways underlying cell proliferation, differentiation, apoptosis, and stress responses. Three parallel MAPK signaling pathways are found in mammalian cells, extracellular signal regulated kinase (ERK), c-Jun N-terminal kinase (JNK), and p38 mitogen-activated protein kinase (p38 MAPK). Inhibiting MAPK signaling can reduce oxidative stress and ensure the normal proliferation, differentiation, and insulin secretion capacity of islet cells^42^. Further, inhibition of the MAPK pathway can improve learning and memory^43^ and suppress apoptosis of hippocampal CA1 neurons by downregulating the transcription factor c-Fos. Activation of the MAPK/ERK pathway may be an important mechanism underlying early and medium-term cognitive impairment in rats, while inhibition can reduce damage and promote the repair of damaged neurons. Quercetin acts on membrane EGFRs and TNFR1 by promoting the release of VEGFA and TNF, thereby affecting the activation of Ras, Raf, MEK, and phosphorylation of MAPK1 and MAPK3, leading to changes in angiogenesis, cell apoptosis, and cell proliferation. More in-depth studies on these mechanisms and preclinical studies in T2DM and AD animal models are warranted to provide a foundation for future clinical studies targeting the MAPK signaling pathway as a novel therapeutic strategy for T2DM and AD.

In conclusion, we have identified several potential molecular mechanisms by which quercetin can simultaneously interfere with T2DM and AD progression. Among these, suppression of MAPK signaling appears to be the most promoting strategy for the treatment of T2DM and AD.

## Supplementary Information

Supplementary Information 1.

Supplementary Information 2.

## Data Availability

The data that supports the findings of this study are available in the supplementary material of this article.
