# Supplementary material for: Mechanism of quercetin therapeutic targets for Alzheimer disease and type 2 diabetes mellitus
Source: Sci Rep. 2021 Nov 25;11:22959. doi: 10.1038/s41598-021-02248-5 (PMC8617296; doi:10.1038/s41598-021-02248-5)
Supplement: Supplementary file 1 — Supplementary Information 1. [file 41598_2021_2248_MOESM1_ESM.pdf]

S1. Quercetin target information table

No.	Target	No.	Target	No.	Target	No.	Target	No.	Target
1	PTGS1	56	CASP8	111	AHR	166	CEBPB	221	ALOX12
2	AR	57	TOP1	112	PSMD3	167	COMT	222	MET
3	PPARG	58	RAF1	113	SLC2A4	168	CSNK2A1	223	NEK2
4	PTGS2	59	SOD1	114	COL8A1	169	CSNK2B	224	CXCR1
5	HSP90	60	PRKCA	115	CXCL11	170	DHFRL1	225	CAMK2B
6	PIK3CG	61	MMP1	116	CXCL2	171	DNMT1	226	ALK
7	NCOA2	62	HIF1A	117	DCAF5	172	EIF3F	227	ABCB1
8	DPP4	63	STAT1	118	NR1I3	173	ESR1	228	NEK6
9	AKR1B10	64	RUNX1T1	119	CHEK2	174	ESR2	229	PLA2G1B
10	PRSS1	65	CDK1	120	INSR	175	ESRRA	230	BACE1
11	TOP2A	66	HSPA5	121	CLDN4	176	ESRRB	231	AXL
12	F2	67	ERBB2	122	PPARA	177	GPER1	232	ABCG2
13	KCNH2	68	ACACA	123	PPARD	178	HCK	233	NUAK1
14	SCN5A	69	HMOX1	124	HSF1	179	HIBCH	234	AKR1C2
15	F10	70	CYP3A4	125	CRP	180	HSP90AA1	235	AKR1C1
16	ADRB2	71	CYP1A2	126	CXCL10	181	HSPA2	236	AKR1C3
17	MMP3	72	CAV1	127	CHUK	182	IGHG1	237	AKR1C4
18	PRKACA	73	MYC	128	SPP1	183	JAK1	238	CA13
19	F7	74	F3	129	RUNX2	184	NCOA1	239	AKR1A1
20	NOS3	75	GJA1	130	RASSF1	185	NQO2	240	GPR35
21	RXRA	76	CYP1A1	131	E2F1	186	PIM1	241	MAPT
22	ACHE	77	ICAM1	132	E2F2	187	PTK2B	242	KDM4E
23	GABRA1	78	IL1B	133	ACP3	188	RUVBL2	243	MYLK
24	MAOB	79	CCL2	134	CTSD	189	SF3B3	244	APEX1
25	RELA	80	SELE	135	IGFBP3	190	SHBG	245	PTPRS
26	EGFR	81	VCAM1	136	IGF2	191	SQLE	246	MPG
27	AKT1	82	PTGER3	137	CD40LG	192	STK17B	247	SLC22A12
28	VEGFA	83	CXCL8	138	IRF1	193	SYK	248	CDK5R1
29	CCND1	84	PRKCB	139	ERBB3	194	UBA1	249	CCNB3
30	BCL2	85	BIRC5	140	PON1	195	UGT3A1	250	ARG1
31	BCL2L1	86	DUOX2	141	DIO1	196	NOX4	251	CDK2
32	FOS	87	HSPB1	142	PCOLCE	197	AVPR2	252	TYR
33	CDKN1A	88	TGFB1	143	NPEPPS	198	AKR1B1	253	HSD17B1
34	EIF6	89	SULT1E1	144	HK2	199	MAOA	254	APP
35	BAX	90	MGAM	145	NKX3-1	200	IGF1R	255	PARP1
36	CASP9	91	IL2	146	RASA1	201	FLT3	256	TTR
37	PLAU	92	NR1I2	147	GSTM1	202	CYP19A1	257	MMP12
38	MMP2	93	CYP1B1	148	GSTM2	203	AURKB	258	CD38
39	MMP9	94	CCNB1	149	ACTB	204	DRD4	259	TNKS2
40	MAPK1	95	PLAT	150	ATP5A1	205	ADORA1	260	TNKS
41	IL10	96	THBD	151	ATP5B	206	GLO1	261	TERT
42	EGF	97	SERPINE1	152	ATP5C1	207	PIK3R1	262	CDK5

43	RB1	98	COL19A1	153	CA1	208	ADORA2A	263	CCNB2
44	TNF	99	IFNG	154	CA12	209	DAPK1		
45	JUN	100	ALOX5	155	CA14	210	PYGL		
46	IL6	101	PTEN	156	CA2	211	GSK3B		
47	CDKN2A	102	IL1A	157	CA3	212	SRC		
48	AHSA1	103	MPO	158	CA4	213	PTK2		
49	CASP3	104	NCF1	159	CA5A	214	HSD17B2		
50	TP53	105	ABCA2	160	CA5B	215	KDR		
51	ELK1	106	HAS2	161	CA6	216	MMP13		
52	NFKBIA	107	GSTP1	162	CA7	217	ALOX15		
53	POR	108	NFE2L2	163	CA9	218	ABCC1		
54	ODC1	109	NQO1	164	CBR1	219	PLK1		
55	XDH	110	TNKS	165	CDK6	220	PKN1		
