# Supplementary material for: Mechanism of quercetin therapeutic targets for Alzheimer disease and type 2 diabetes mellitus
Source: Sci Rep. 2021 Nov 25;11:22959. doi: 10.1038/s41598-021-02248-5 (PMC8617296; doi:10.1038/s41598-021-02248-5)
Supplement: Supplementary file 2 — Supplementary Information 2. [file 41598_2021_2248_MOESM2_ESM.pdf]

S2 Quercetin and Alzheimer's disease and type 2 diabetes coincide with targets

No.	Target	No.	Target	No.	Target
1	PTGS1	33	NFKBIA	65	PPARA
2	AR	34	POR	66	PPARD
3	PPARG	35	XDH	67	CRP
4	PTGS2	36	CASP8	68	CXCL10
5	PIK3CG	37	SOD1	69	SPP1
6	PRSS1	38	MMP1	70	IGFBP3
7	F2	39	HMOX1	71	IGF2
8	F10	40	CYP3A4	72	CD40LG
9	ADRB2	41	CYP1A2	73	PON1
10	MMP3	42	CAV1	74	GSTM1
11	F7	43	F3	75	ATP5B
12	NOS3	44	CYP1A1	76	CA1
13	RXRA	45	ICAM1	77	CA2
14	ACHE	46	IL1B	78	CA4
15	MAOB	47	CCL2	79	COMT
16	EGFR	48	SELE	80	ESR1
17	AKT1	49	VCAM1	81	ESR2
18	VEGFA	50	PRKCB	82	SHBG
19	BCL2	51	TGFB1	83	AKR1B1
20	BAX	52	IL2	84	IGF1R
21	CASP9	53	PLAT	85	CYP19A1
22	PLAU	54	THBD	86	ADORA1
23	MMP2	55	SERPINE1	87	GLO1
24	MMP9	56	IFNG	88	PIK3R1
25	MAPK1	57	PTEN	89	KDR
26	IL10	58	IL1A	90	ALOX12
27	TNF	59	MPO	91	MAPT
28	JUN	60	GSTP1	92	APP
29	IL6	61	NQO1	93	PARP1
30	CDKN2A	62	AHR	94	TTR
31	CASP3	63	SLC2A4	95	CDK5
32	TP53	64	INSR		
